# Effects of movement training based on rhythmic auditory stimulation in cognitive impairment: a meta-analysis of randomized controlled clinical trial

**DOI:** 10.3389/fnins.2024.1360935

**Published:** 2024-04-15

**Authors:** Ya Nan Wang, Xiao Ni Wen, Yu Chen, Nuo Xu, Jing Han Zhang, Xue Hou, Jing Ping Liu, Ping Li, Jia Yu Chen, Jun Hao Wang, Xin Yue Sun

**Affiliations:** ^1^Xi'an Physical Education University, Xi'an, China; ^2^School of Exercise and Health Sciences, Xi'an Physical Education University, Xi'an, China

**Keywords:** cognitive impairment, movement training based on rhythmic auditory stimulation, aging, meta-analysis, randomized controlled trial

## Abstract

**Objective:**

According to the World Alzheimer’s Disease Report in 2015,there were 9.9 million new cases of dementia in the world every year. At present, the number of patients suffering from dementia in China has exceeded 8 million, and it may exceed 26 million by 2040.Mild cognitive impairment (MCI) refers to the pathological state of pre-dementia with the manifestation of the progressive decline of memory or other cognitive functions but without decline of activities of daily life. It is particularly important to prevent or prolong the development of MCI into dementia. Research showing effects of rhythmic auditory stimulation based-movement training(RASMT) interventions on cognitive function is also emerging. Therefore, the present meta-analysis briefly summarize findings regarding the impacts of RASMT programs on cognitive impairment.

**Methods:**

Data from Pubmed, Embase, and Cochrane Library were utilized. The impact of RASMT on cognitive functions was evaluated using indicators such as overall cognitive status, memory, attention, and executive functions. The REVMAN5.3 software was employed to analyze bias risks integrated into the study and the meta-analysis results for each indicator.

**Results:**

A total of 1,596 studies were retrieved, of which 1,385 non-randomized controlled studies and 48 repetitive studies were excluded. After reviewing titles and abstracts of the remaining 163 articles, 133 irrelevant studies were excluded, 30 studies were downloaded and read the full text. Among 30 articles, 18 articles that did not meet the inclusion criteria were excluded, the other 12 studies were included in this meta-analysis. Utilizing the Cochrane Collaborative Network Bias Risk Assessment Scale, it was found that 11 studies explained the method of random sequence generation, nine studies did not describe allocation concealment, four were single-blinded to all researchers, and eight reported single-blinding in the evaluation of experimental results. In the meta-analysis, the main outcomes showed statistically significant differences in overall cognitive status [MD = 1.19, 95%CI (0.09, 2.29), (*p* < 0.05)], attention [MD = −1.86, 95%CI (−3.53, −0.19), (*p* < 0.05)], memory [MD = 0.71, 95%CI (0.33, 1.09), (*p* < 0.01)], and executive function [MD = −0.23, 95% CI (−0.44, −0.02), (*p* < 0.05)]. Secondary outcomes indicated no statistically significant differences in verbal fluency [MD = −0.51, 95%CI (−1.30, 0.27), (*p* = 0.20)], while depression [MD = −0.29, 95% CI (−0.42, −0.16), (*p* < 0.01)] and anxiety [MD = 0.19, 95% CI (0.06, 0.32), (*p* < 0.01)] exhibited statistically significant differences. The GRADEpro GDT online tool assessed the quality of evidence for the outcome measures, revealing one low-quality outcome, two moderate-quality outcomes, and one high-quality outcome in this review.

**Conclusion:**

This study shows that RASMT can improve the general cognitive status, memory, attention and executive function of patients with cognitive impairment. The quality of evidence revealed that MMSE was low, attention and memory were moderate, and executive function was high. The RAMST program (type of exercise: play percussion instruments; time of exercise: 30–60 min; frequency of exercise: 2–3 times/week; duration of exercise: more than 12 weeks) was proved to be more effective in improving cognitive function. However, the sample size is relatively insufficient, the future needs further study.

**Systematic review registration:**

PROSPERO, identifier: CRD42023483561.

## Introduction

Cognition is the intricate process involving the acquisition, encoding, manipulation, extraction, and utilization of sensory input information by individuals. It constitutes a mental process situated between input and output. Cognitive impairment arises when various factors lead to the impairment of memory, attention, language, and executive function, thereby affecting an individual’s capacity to engage in daily or social activities (Woodford and George, 2007). Cognitive impairment is particularly prevalent among older people and is closely associated with the aging process. Epidemiological studies have disclosed that the prevalence of mild cognitive impairment (MCI) is 15.4% in individuals aged 55 years and above in China (Deng et al., 2021), 27.3% in those aged 65 years and above in the United States (Rajan et al., 2021), and 15.56% in those aged 50 years and above globally. Notably, the incidence of MCI tends to rise with advancing age (Bai et al., 2022). Simultaneously, cognitive impairment is often concurrent with certain diseases. In Parkinson’s disease (PD) patients, cognitive impairment is a common cognitive state, with a prevalence of MCI at 40% (Baiano et al., 2020). Post-stroke cognitive impairment (PSCI) emerges as a prevalent functional impairment following a stroke, reaching an incidence rate of up to 80.97% (Qu et al., 2015). This not only impacts the functional recovery of patients and extends the disease course but also elevates the recurrence and mortality rates of stroke (Oksala et al., 2009; Kwon et al., 2020; Kwan et al., 2021). It is estimated that over the next 50 years, the number of older people in the world will increase by 21%, and the elderly population in developing and developed countries will increase by 140 and 51%, respectively (Eshkoor et al., 2015). As population aging intensifies, MCI will become a major threat to the health of the elderly. In addition, a meta-analysis of 41 cohort studies found that the cumulative proportion of MCI cases progressing to dementia was 39.2 percent (Mitchell and Shiri-Feshki, 2009), and treatment of MCI was seen as a potential intervention to slow the progression of dementia (Anderson, 2019). Consequently, the restoration of cognitive function has become a focal point for researchers worldwide. Neurologic music therapy (NMT) (Impellizzeri et al., 2020), exercise therapy (Swatridge et al., 2017), and virtual reality (Liao et al., 2020) have been implemented in clinical practice to train cognitive function, demonstrating promising efficacy.

NMT, a novel music therapy technique rooted in neurology (de L’Etoile Shannon, 2016), targets cognitive, sensory, and motor dysfunction resulting from neurological disorders in humans. It finds applications in neurological rehabilitation, neuropediatric therapy, neurogeriatric therapy, and neurodevelopmental therapy (Galińska, 2015). A specific therapeutic technique within NMT, rhythmic auditory stimulation (RAS), enhances cognitive function by engaging the basal ganglia-thalamic-cortical network (Kotz et al., 2009; Schwartze et al., 2011) and the cerebellar- thalamus- cortical circuit (Coull et al., 2011; Kotz and Schmidt-Kassow, 2015) through rhythmic and repetitive auditory stimulation. RASMT involves subjects following rhythmic auditory cues (such as music or a metronome) and executing rhythmic movements (e.g., playing percussion, piano, clapping, walking) involving the upper limbs, lower limbs, and trunk. This method concurrently conveys visual, auditory, and motoric information to a specialized brain network comprising fronto-temporo-parietal regions, components of the putative human mirror neuron system. This network, among its various functions, likely facilitates the coupling between perceptual events (visual or auditory) and motor actions (leg, arm/hand, or vocal/articulatory actions), integrating cognition and perception to enhance cognitive function (Schlaug et al., 2010). Research indicates that movement training with musical rhythm can stimulate the prefrontal cortex and enhance overall cognitive status in healthy older people with MCI compared to movement training without musical rhythm (Shimizu et al., 2018). This finding is further supported by Hars et al. (2014), who assessed Frontal Assessment Battery (FAB) and Mini-Mental State Examination (MMSE) scores before and after 6 months of walking to the rhythm of the piano in older people with MCI. Furthermore, RASMT exhibits positive effects on memory, attention, and executive function. Studies demonstrate that playing instrument training can improve memory and overall cognitive status in MCI patients (Doi et al., 2017). Drum playing intervention with rhythmic cueing (DPRC) has great potential to improve upper limb motor control and attention control (Park and Kim, 2021). Ronnie Gardner’s rhythm and music (RGRM) training have been shown to improve learning and episodic memory, speed, attention, visuospatial functions, language, and executive functions in Parkinson’s disease patients (Pohl et al., 2013, 2020). Auditory motor synchronization training (AMST) has been found to enhance attention, memory, and executive function in stroke patients (Park and Lee, 2018).

In conclusion, RASMT not only proves beneficial for addressing cognitive impairment in the normal aging process but also for the improvement of cognitive impairment in various diseases. However, it is noteworthy that as of now, in previous studies when discussing the effects of RASMT on cognitive impairment, there were differences in the subjects (including healthy people, mild to severe cognitive impairment, stroke, Parkinson’s disease, etc.), the intervention methods (specific exercise forms, exercise time, exercise frequency, duration) and the research objectives (the effects of RASMT on the general cognitive function, such as memory, attention, executive ability, speech fluency, etc.). To explore whether differences in subjects and intervention methods affect RAMST’s effects on cognitive function, and which aspects of RAMST’s improvement in cognitive function are more significant, we conducted a meta-analysis of randomized controlled trials. The selected studies for inclusion in this meta-analysis adhere to the criteria of being randomized controlled trials, ensuring a higher standard of research methodology. This rigorous selection aims to enhance the quality of the articles under examination, thereby furnishing more precise and evidence-based insights for the clinical treatment of cognitive impairment.

## Methods

In this endeavor, a systematic review was meticulously planned and executed, adhering to the guidelines outlined in the Preferred Reporting Items for Systematic Reviews and Meta-Analyses (PRISMA) statement. Additionally, this review was duly registered with PROSPERO under the registration number CRD42023483561.

### Search strategy

Two reviewers conducted an electronic search of the Pubmed, Embase, and Cochrane Library databases in November 2023. The search utilized the following keyword combinations: rhythmic auditory stimulation, rhythmic auditory cues, music therapy, movement music therapy, rhythm and music-based therapy, active music therapy, and cognitive impairment. Additionally, these key terms were meticulously matched with the relevant Medical Subject Headings (MeSH) terms. Pre-searches were conducted, and the final search strategy was determined as follows: PUBMED: (((((Music Therapy[MeSH Terms]) OR (rhythmic cueing[Title/Abstract])) OR (rhythmic auditory stimulation[Title/Abstract])) OR (movement music therapy[Title/Abstract])) OR (rhythm music-based therapy[Title/Abstract])) OR (active music therapy[Title/Abstract]) AND ((((((((((((((((((((((((Cognitive Dysfunction[MeSH Terms]) OR (cognitive dysfunctions[Title/Abstract])) OR (dysfunction, cognitive[Title/Abstract])) OR (dysfunctions, cognitive[Title/Abstract])) OR (cognitive impairments[Title/Abstract])) OR (cognitive impairment[Title/Abstract])) OR (impairment, cognitive[Title/Abstract])) OR (impairments, cognitive[Title/Abstract])) OR (cognitive disorder[Title/Abstract])) OR (cognitive disorders[Title/Abstract])) OR (disorder, cognitive[Title/Abstract])) OR (disorders, cognitive[Title/Abstract])) OR (mild cognitive impairment[Title/Abstract])) OR (cognitive impairment, mild[Title/Abstract])) OR (cognitive impairments, mild[Title/Abstract])) OR (impairment, mild cognitive[Title/Abstract])) OR (impairments, mild cognitive[Title/Abstract])) OR (mild cognitive impairments[Title/Abstract])) OR (cognitive decline[Title/Abstract])) OR (cognitive declines[Title/Abstract])) OR (decline, cognitive[Title/Abstract])) OR (declines, cognitive[Title/Abstract])) OR (mental deterioration[Title/Abstract])) OR (deteriorations, mental[Title/Abstract])) OR (mental deteriorations[Title/Abstract]).Embase: (‘music therapy’/exp. OR (‘music therapy’:ab,ti OR ‘rhythmic cueing’:ab,ti OR ‘rhythmic auditory stimulation’:ab,ti OR ‘movement music therapy’:ab,ti OR ‘rhythm music-based therapy’:ab,ti OR ‘active music therapy’:ab,ti)) AND (‘cognitive defect’/exp. OR (‘cognition disorder’:ab,ti OR ‘cognition disorders’:ab,ti OR ‘cognitive defects’:ab,ti OR ‘cognitive deficit’:ab,ti OR ‘cognitive disability’:ab,ti OR ‘cognitive disorder’:ab,ti OR ‘cognitive disorders’:ab,ti OR ‘cognitive dysfunction’:ab,ti OR ‘cognitive impairment’:ab,ti OR ‘cognitive defect’:ab,ti)). Cochrane Library:(MeSH descriptor: [Music Therapy] explode all trees OR (rhythmic cueing:ti,ab OR (rhythmic auditory stimulation:ti,ab OR (movement music therapy:ti,ab OR (rhythm music-based therapy:ti,ab OR (active music therapy:ti,ab)) AND (MeSH descriptor: [Cognitive Dysfunction] explode all trees OR (cognitive dysfunctions:ti,ab OR (dysfunction, cognitive:ti,ab OR (dysfunctions, cognitive:ti,ab OR (cognitive impairments:ti,ab OR (cognitive impairment:ti,ab OR (impairment, cognitive:ti,ab OR (impairments, cognitive:ti,ab OR (cognitive disorder:ti,ab OR (cognitive disorders:ti,ab OR (disorder, cognitive:ti,ab OR (disorders, cognitive:ti,ab OR (mild cognitive impairment:ti,ab OR (cognitive impairment, mild:ti,ab OR (cognitive impairments, mild:ti,ab OR (impairment, mild cognitive:ti,ab OR (impairments, mild cognitive:ti,ab OR (mild cognitive impairments:ti,ab OR (cognitive decline:ti,ab OR (cognitive declines:ti,ab OR (decline, cognitive:ti,ab OR (declines, cognitive:ti,ab OR (mental deterioration:ti,ab OR (deteriorations, mental:ti,ab OR (mental deteriorations:ti,ab)).Meanwhile, a manual search (an online search of relevant journals and references of review articles) was conducted to identify papers that may have been missed in the electronic database search.

### Eligibility criteria

#### Inclusion criteria

The Population, Intervention, Comparison, Outcomes, Study Design (PICOS) framework (Cristini et al., 2021) was employed to establish the eligibility criteria for articles included in this review. Within this meta-analysis, the populations encompassed studies involving participants diagnosed with cognitive impairment. Interventions comprised studies utilizing RASMT as an experimental group intervention, with a clearly defined protocol encompassing specific training parameters (exercise form, exercise time, exercise frequency, and duration). To facilitate comparison with the experimental group, included studies necessitated an intervention in the control group (such as conventional treatment, music listening, cognitive training, etc.). Results encompassed both main and secondary outcomes. Main outcomes focused on the study of overall cognitive status, attention, memory, and executive function. Evaluation methods included the MMSE, FAB, Trail Making Test (TMT), and Digit Span Test (DST). Secondary outcomes delved into the assessment of word fluency, depression and anxiety. Evaluation methods for secondary outcomes encompassed the Verbal Fluency Test (VFL), Hospital Anxiety and Depression Scale (HADS), and Near-Infrared Spectroscopy (NIRS). Word fluency, depression and anxiety are also manifestations of cognitive impairment. NIRS, as a functional neuroimaging modality characterized by non-invasiveness and high mobility, can quantitatively measure the concentration of hemoglobin in brain tissue to assess oxygen metabolism, indirectly reflecting neuronal activity in the brain. Therefore we included VFL, HADS and NIRS as secondary assessment measures to more comprehensively and accurately assess the effect of RASMT on participants’ cognitive function. Study Design strictly encompassed Randomized Controlled Trials (RCTs), ensuring a robust and high-quality selection for this review.

#### Exclusion criteria

All non-research studies, non-randomized control experiments, animal experiments, and duplicate papers were excluded from consideration. The two reviewers independently assessed the titles and abstracts, initially identifying literature that met the predefined standards. Subsequently, the full text of these selected documents was thoroughly examined to further ascertain their compliance with the established criteria, and data with incomplete information were systematically excluded. The comprehensive screening process adhered to the structured framework outlined in PICOS.

#### Study selection

Initially, the final search results from Pubmed, Embase, and Cochrane Library databases were retrieved individually. The search results excluding non-randomized control experiments were imported into EndNote software, and duplicate studies were subsequently removed. Subsequently, the two authors independently reviewed the titles and abstracts, identifying potentially relevant research. Finally, all potentially relevant studies were downloaded and assessed in detail according to the PICOS principle, ultimately determining the studies that met the established standards. In cases where there was inconsistency between the assessments of the two authors, a senior researcher joined the discussion to facilitate a consensus and reach a final decision.

### Data extraction

The meta-analysis incorporated general information and outcome indicators from the research. General information encompassed the first author, sample size, gender, age, treatment, and intervention measures. Results included primary indicators (overall cognitive status, attention, memory, and executive function) and secondary indicators (verbal fluency, depression and anxiety). Both authors independently extracted and analyzed the data from the included studies. In cases where a study meeting the inclusion criteria lacked effective data, attempts were made to contact the study’s author for original data. If the data remained unavailable or invalid, the study was excluded from the subsequent analysis. In instances where there was a discrepancy in the analysis results between the two authors, a senior researcher joined the discussion to facilitate reaching a final consensus.

### Quality and risk of bias assessment

Utilize the Cochrane bias risk assessment tool (Choi et al., 2020) to evaluate the risk of bias incorporated into the research. Cochrane bias risk assessment tools address six aspects, namely random sequence generation (selection bias), allocation concealment (selection bias), blinding of participants and personnel (performance bias), blinding of outcome assessment (detection bias), incomplete outcome data (attrition bias), and selective reporting (reporting bias). Each aspect is assessed against three standards: high risk, unclear risk, and low risk. Following this assessment, the RevMan 5.3 software was employed to generate a bias risk chart for this meta-analysis.

Assess the quality of evidence using the GRADEpro GDT online tool for outcome indicators. GRADE, a rating system proposed by a working group comprising over 60 doctors, clinical epidemiologists, and evidence-based medical experts in 2000, currently serves as the standard for evaluating evidence quality. The assessment of risk of bias, inconsistency, indirectness, imprecision, and publication bias categorizes the quality of evidence into four levels: high, medium, low, and extremely low quality.

Both of the aforementioned evaluations are independently conducted by the two authors. In cases where there is inconsistency in the evaluation results between the two authors, a senior researcher will participate in the discussion to reach a final consensus.

### Statistical analysis

Utilize RevMan 5.3 (Cochrane Collaboration Network, Oxford, Britain) for statistical analysis. In the statistical analysis of continuous variables’ average and standard deviation, a significance level of *p* < 0.05 in the results indicates that the overall effect (Z) holds statistically significant importance. If I^2^ is less than 50%, it suggests insignificant heterogeneity, and a fixed-effect model is applied. Conversely, if I^2^ exceeds 50%, it implies significant heterogeneity. Sub-group meta-analyses and sensitivity analysis are conducted to identify the source of heterogeneity. If heterogeneity persists, a random-effect model is employed for the summary analysis (Choi et al., 2020).

## Results

### Search results

**Figure 1:**
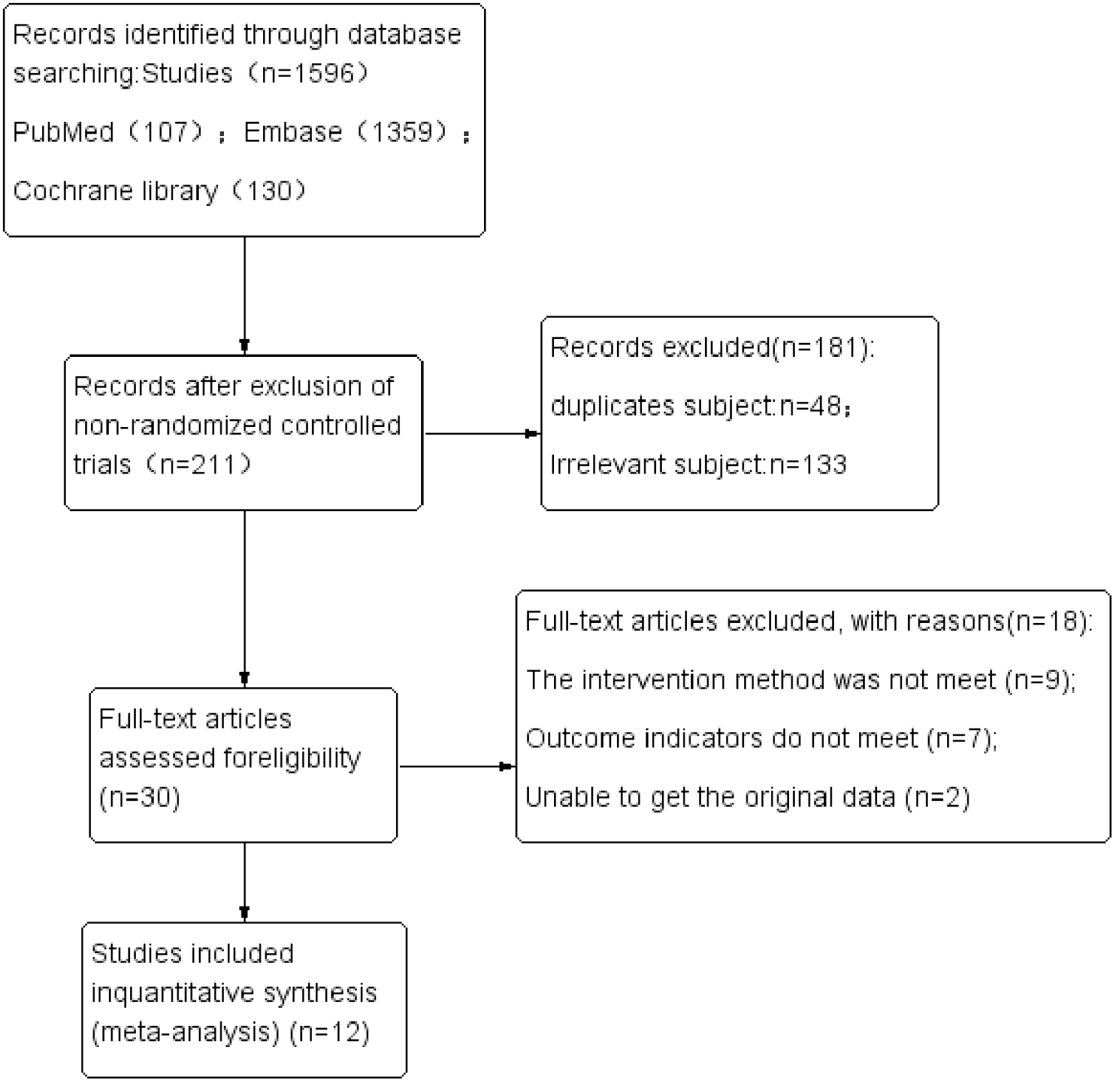
Flow chart of the search process.

The three primary databases—Pubmed, Embase, and Cochrane Library—yielded a total of 107, 1,359, and 130 studies, respectively, amounting to 1,596 studies in total. After excluding non-random control experiments, 1,385 studies were eliminated, leaving 211 studies. Among these, 35 were from Pubmed, 49 from Embase, and 127 from Cochrane Library. After importing into Endnote software and excluding 48 duplicate studies, the remaining 163 titles and abstracts were scrutinized, resulting in the elimination of 133 unrelated documents. Subsequently, the remaining 30 studies were downloaded, and following a thorough examination of the full text, 18 studies that did not meet the standards were removed. In the end, the meta-analysis was conducted with 12 studies. For specific details and steps, refer to Figure 1.

### Quality and risk of bias assessment

**Figure 2:**
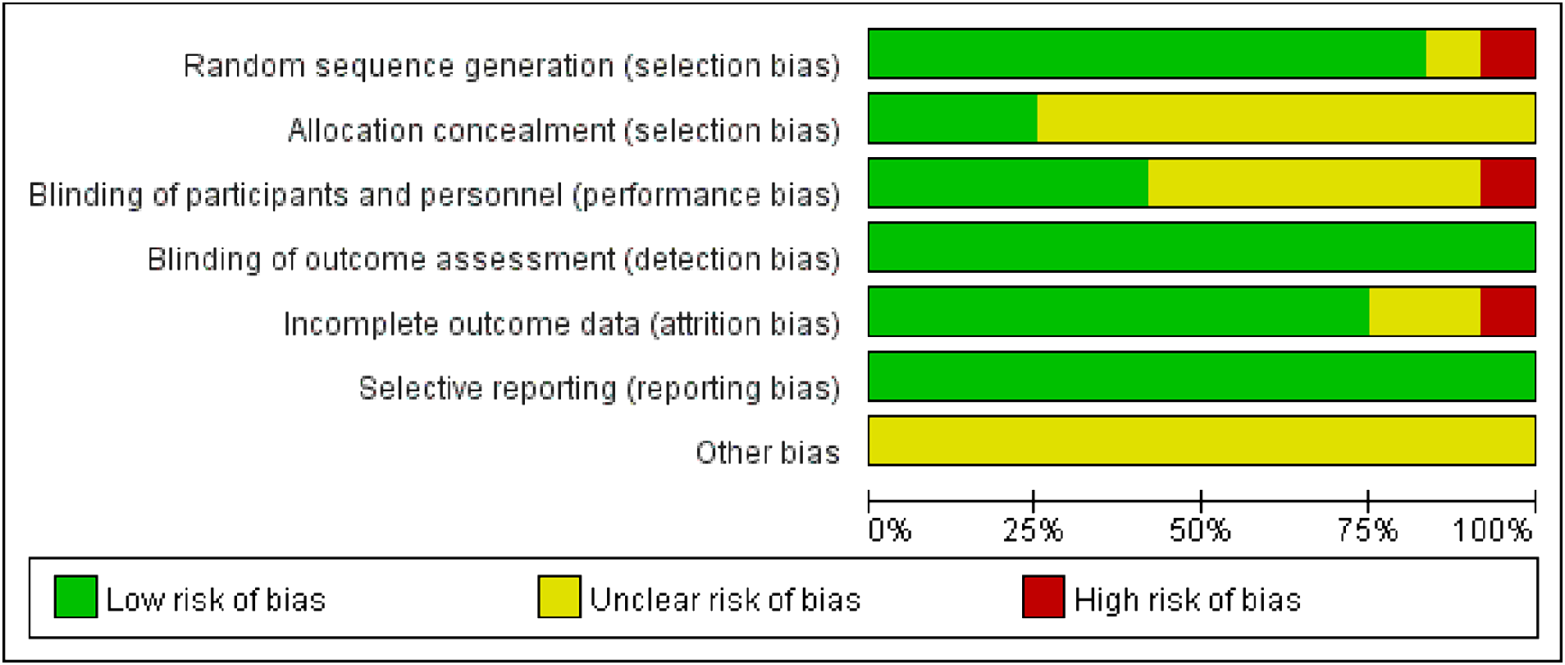
Risk of bias graph.

**Figure 3:**
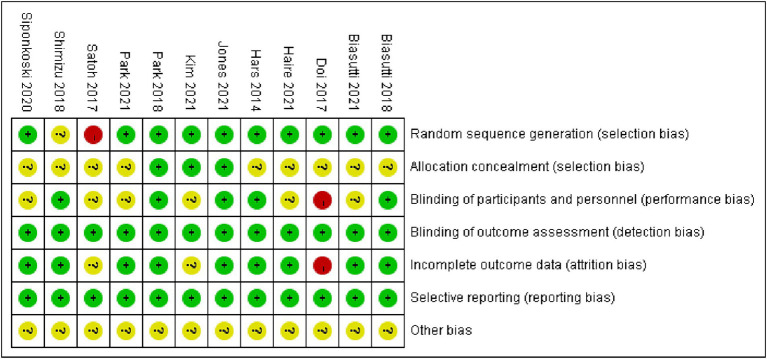
Risk of bias summary.

Utilize the Cochrane Collaborative Network Bias Risk Assessment Scale for bias evaluation, as illustrated in Figures 2, 3. A total of 12 studies underwent evaluation, revealing that 11 studies detailed the method of employing random sequences. However, one study utilized the floor and ward number for randomization and did not provide a clear report of the random sequence. Allocation concealment was not described in nine studies, and only four studies implemented a blind method for all researchers. Eight studies reported the assessment of experimental results without implementing a blind method for researchers, but this had minimal impact on the research outcomes. All studies demonstrated low risk of bias for attrition and reporting. Please refer to Figures 2, 3 for a detailed representation.

### Study characteristics

The general information encompassed in the research, detailed in Table 1, consists of the first author, sample size, gender, age, and diagnostic standards. The data incorporated into the study, outlined in Table 2, includes the intervention schemes for both the experimental and control groups. This involves the form of movement, duration, frequency, and time of RASMT, along with the assessment time points and measurement outcomes. Within the included research, the smallest sample size is 15 (Street et al., 2015), while the largest is 134 (Hars et al., 2014; Doi et al., 2017). Various movement forms are implemented in the experimental groups, such as intensive cognitive music training, playing percussion instruments, structured music-based multitask exercise classes, rhythmic intervention music training, and physical exercise with music—all falling under the umbrella of RASMT. Control group interventions encompass a range of activities, including gymnastics, health education, conventional activities, computer cognitive training, and more. The intervention durations vary significantly, ranging from 3 weeks to 40 weeks.

**Table 1:** Characteristics of participants in the included studies.

Study	Gender(F/M)	Age(year)	Sample(n)	MMSE
	C/E	C/E	C/E	C/E
Biasutti and Mangiacotti (2018)	9/8	83.76 ± 6.16	17/18	24.53 ± 3.50
	14/4	83.39 ± 7.81		22.39 ± 3.65
Biasutti and Mangiacotti (2021)	14/11	85.12 ± 6.14	25/20	23.64 ± 4.34
	15/5	83.95 ± 7.84		22.45 ± 3.46
Doi et al. (2017)	31/36	76.0 ± 4.9	67/67	25.8 ± 2.4
	39/28	76.2 ± 4.6		25.9 ± 2.6
Haire et al. (2021)	6/4	57.6 ± 11.14	10/10	
	5/5	55.5 ± 15.01		
Hars et al. (2014)	65 /96	76 ± 6	68/66	26.3 ± 3.0
	64/97	75 ± 8		25.9 ± 2.7
Jones et al. (2021)	1/6	55.4 ± 10.54	7/8	
	1/7	51.9 ± 11.02		
Kim and Kang (2021)	17/3		20/20	
	14/6			
Park and Lee (2018)	6/7		15/5	
	7/8			
Park and Kim (2021)		63.1 ± 10.1	8/8	26.3 ± 1.9
		61.6 ± 4.9		28.3 ± 1.2
Satoh et al. (2017)		87.4 ± 4.4	31/31	20.9 ± 3.72
		87 ± 5.4		20.1 ± 3.17
Shimizu et al. (2018)	10/1	73.33 ± 7.31	9/30	
	28/6	74.90 ± 4.29		
Siponkoski et al. (2020)	3/6	40.8 ± 11.5	20/20	
	7/9	42.1 ± 14.6		

C, control group; E, experiment group; M, male; F, female.

**Table 2:** Characteristics of study design in the included studies.

Study	Interventions(C)	Interventions(E)	Methods, minutes, frequency, duration	Assessment time points	Outcome measures
Biasutti and Mangiacotti (2018)	Gymnastic activities	RASMT(intensive cognitive music training)	45 min, 1time/2 week, 24 weeks	After 4 months of treatment	MMSE, VFL, TMT-A, AMT, CDT
Biasutti and Mangiacotti (2021)	Gymnastic activities	RASMT(intensive cognitive music training)	70 min, 2times/week, 6 weeks	After 6 weeks of treatment	MMSE, GDS
Doi et al. (2017)	Health education	RASMT(playing percussion instruments)	60 min, 1time/week, 40 weeks	After 40 weeks of treatment	Story memory, Word memory MMSE, TMT(A B)
Haire et al. (2021)	TIMP+MI	RASMT(TIMP+cMI)	45 min, 3times/week, 3 weeks	After 3 weeks of treatment	TMT(B), DST, MAACL-R, GSE, SAM
Hars et al. (2014)	Maintained usual lifestyle habits,	RASMT(structured music-based multitask exercise classes)	60 min,1 time/week, 6 months	After 6 months of treatment	MMSE, CDT, FAB, HADS-A
Jones et al. (2021)	Attention process training	RASMT(music attention control training)	45 min, 1time/week, 3 weeks	After 3 weeks of treatment	TMT(A B), DST, BPT
Kim and Kang (2021)	Regular activities	RASMT(music intervention with rhythmic exercises+regular activities)	50 min,1 time/2 weeks, 12 weeks	After 12 weeks of treatment	MMSE, GDSSF-K, GAI-K, Life satisfaction
Park and Lee (2018)	CMDT	RASMT(CMDT+AMST)	30 min, 3times/week, 6 weeks	After 6 weeks of treatment	TMT, DST, ST
Park and Kim (2021)	Regular programs	RASMT(drum playing intervention with rhythmic cueing)	50 min, 3times/week, 12 weeks	After 12 weeks of treatment	NHPT, TMT(A B), KST
Satoh et al. (2017)	Cognitive stimulation	RASMT(physical exercise with music)	40 min, 1time/week, 6 months	After 6 months of treatment	MMSE, TMT(A), RCPM, RBMT, WF
Shimizu et al. (2018)	Single-training task	RASMT(movement music therapy)	50 min, 1time/week, 12 weeks	After 12 weeks of treatment	FAB, NIRS
Siponkoski et al. (2020)	Standard care	RASMT(neurological music therapy+standard care)	60 min, 2times/week, 3 months	After 3 months of treatment	FAB, Executive function, Reasoning, Verbal memory, Motor performance

MMSE, Mini-Mental State Examination; VFL, verbal fluency test; CDT, clock drawing test; TMTA/B, Trail Making Test A/B; AMT, attentional matrices test; GDS, Geriatric Depression Scale; TIMP, Therapeutic Instrumental Music Performance; MI, Motor Imagery; cMI, metronome-cued motor imagery; MAACL-R, Multiple Affect Adjective Checklist; GSE, General Self-Efficacy scale; SAM, Self-Assessment Manikin; FAB, frontal assessment battery; HADS, hospital anxiety and depression scale; BPT, Brown-Peterson Task; GDSSF-K, Geriatric Depression Scale; GAI-K, Geriatric Anxiety Inventory-Korean; CMDT, cognitive-motor dual-task training; AMST, auditory motor synchronization training; ST, Stroop test; NHPT, Nine Hole Peg Test; KST, Korean Stroop test; RCPM, Japanese Raven’s Colored Progressive Matrices; RBMT, Rivermead Behavioral Memory Test; WF, word fluency; NIRS, near-infrared spectroscopy.

### Outcomes

#### Main outcome

**Figure 4:**
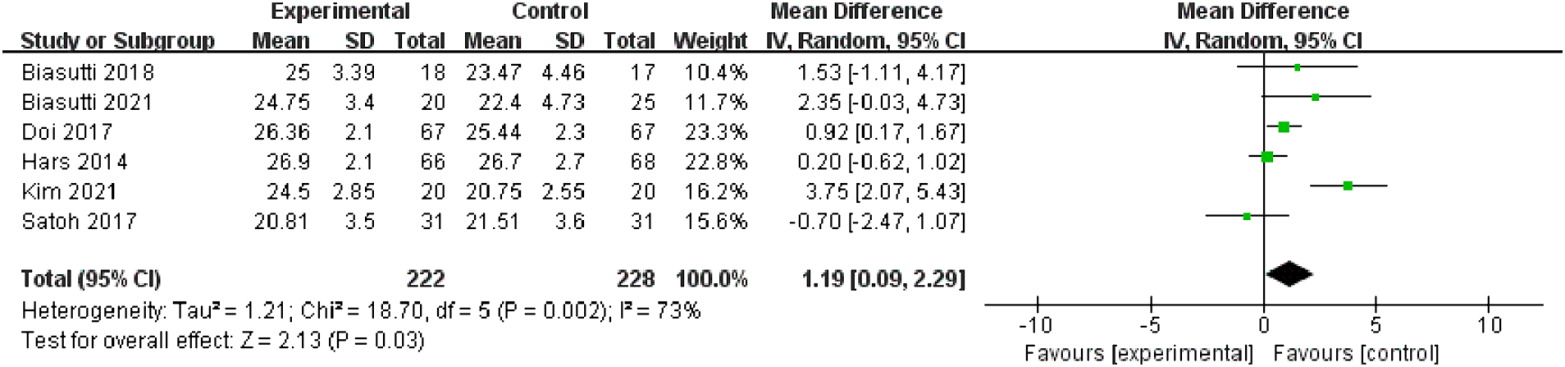
Forest plot of overall cognitive status for meta-analysis.

**Figure 5:**
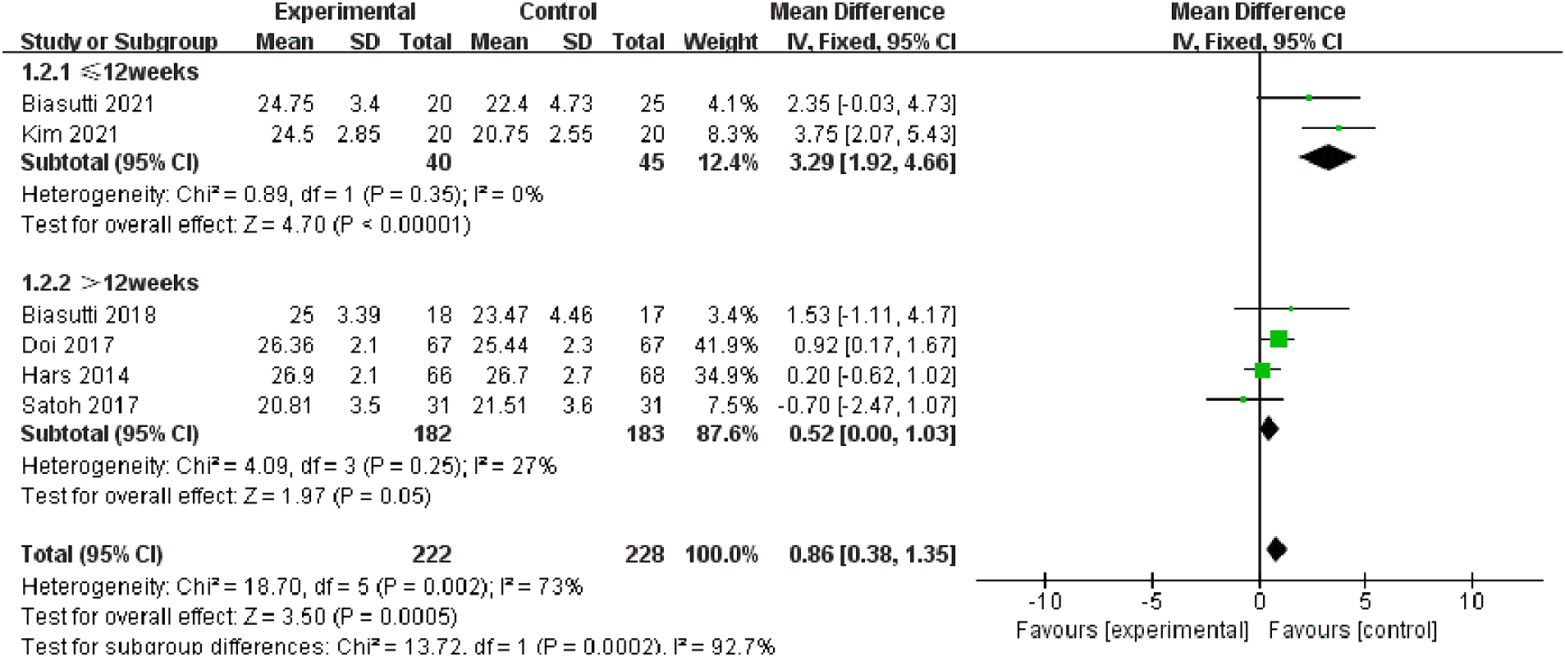
Forest plot for subgroup analysis.

Overall cognitive status: Six studies, incorporating 450 subjects, were included in the analysis of overall cognitive status. The results revealed significant heterogeneity (I^2^ = 73%), prompting the adoption of a random-effect model [MD = 1.19, 95% CI (0.09, 2.29), (*p* < 0.05)]. Subsequently, a sub-group analysis was conducted, categorizing the intervention duration as ≤12 weeks and > 12 weeks. In the sub-group with ≤12 weeks, comprising two studies, results indicated non-significant heterogeneity (*p* = 0.35, I^2^ = 0%), thus a fixed effect model was chosen [MD = 3.29, 95% CI (1.92, 4.66), (*p* < 0.01)]. For the >12 weeks sub-group, consisting of four studies, results also demonstrated non-significant heterogeneity (*p* = 0.25, I^2^ = 27%), leading to the selection of a fixed effect model (*p* = 0.05). Consequently, the sub-group analysis yielded statistically significant results (Figures 4, 5).

**Figure 6:**
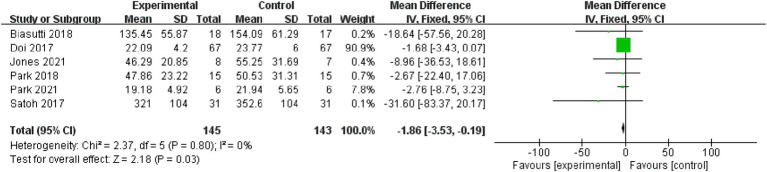
Forest plot of attention for meta-analysis.

Attention: Six studies, involving 288 subjects, were included in the attention analysis. Results indicated non-significant heterogeneity (*p* = 0.8, I^2^ = 0%), leading to the use of a fixed effect model [MD = −1.86, 95% CI (−3.53, −0.19), (*p* < 0.05)]. Therefore, the difference between the two groups was statistically significant (Figure 6).

**Figure 7:**
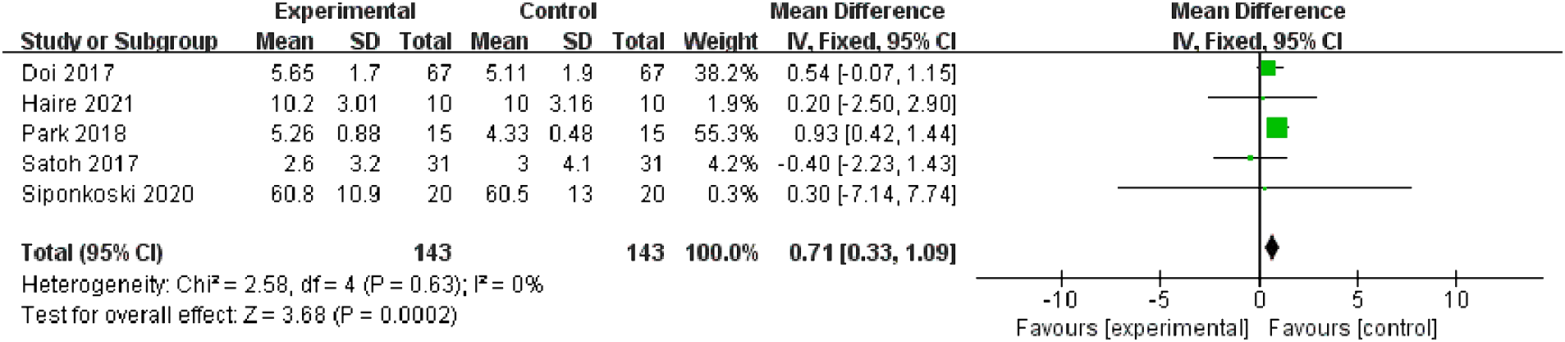
Forest plot of memory for meta-analysis.

Memory: Five studies, encompassing 286 subjects, were included in the memory analysis. Non-significant heterogeneity was observed (*p* = 0.63, I^2^ = 0%), prompting the application of a fixed effect model [MD = 0.71, 95% CI (0.33, 1.09), (*p* < 0.01)]. Consequently, the difference between the two groups was statistically significant (Figure 7).

**Figure 8:**
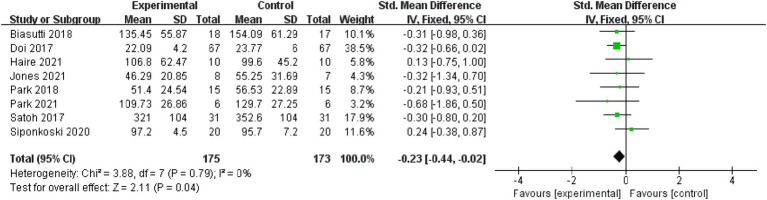
Forest plot of executive function for meta-analysis.

Executive function: Eight studies, with 348 subjects, were included in the analysis of executive functions. Results indicated non-significant heterogeneity (*p* = 0.79, I^2^ = 0%), leading to the use of a fixed effect model [MD = −0.23, 95% CI (−0.44, −0.02), (*p* < 0.05)]. Therefore, the difference between the two groups was statistically significant (Figure 8).

#### Secondary outcome

**Figure 9:**
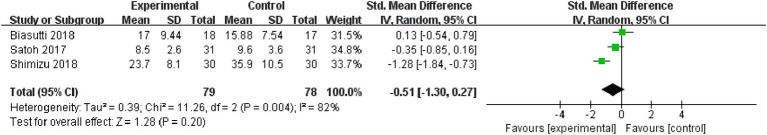
Forest plot of verbal fluency for meta-analysis.

Verbal fluency: The analysis on word fluency includes two studies, involving 97 subjects. The results indicate significant heterogeneity (*p* = 0.004, I^2^ = 82%). Consequently, a random-effect model was employed [MD = −0.51, 95% CI (−1.30, 0.27), (*p* = 0.20)]. Hence, the difference between the two groups is not statistically significant (Figure 9).

**Figure 10:**
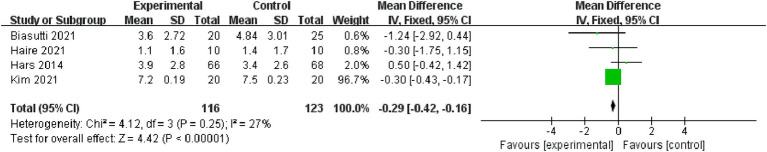
Forest plot of depression for meta-analysis.

Depression: Four studies, encompassing 239 subjects, were incorporated into the depression analysis. The results reveal non-significant heterogeneity (*p* = 0.25, I^2^ = 27%), prompting the use of a fixed effect model [MD = −0.29, 95% CI (−0.42, −0.16), (*p* < 0.01)]. Therefore, the differences between the two groups are statistically significant (Figure 10).

**Figure 11:**
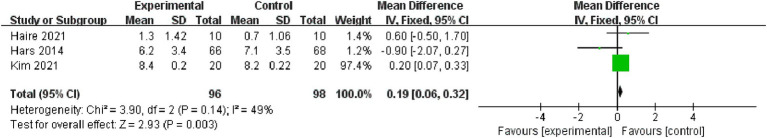
Forest plot of anxiety for meta-analysis.

Anxiety: The analysis on anxiety includes three studies, involving 194 subjects. The results demonstrate non-significant heterogeneity (*p* = 0.14, I^2^ = 49%), leading to the selection of a fixed effect model [MD = 0.19, 95% CI (0.06, 0.32), (*p* < 0.01)]. Hence, the difference between the two groups holds statistical significance (Figure 11).

### Grade

**Figure 12:**
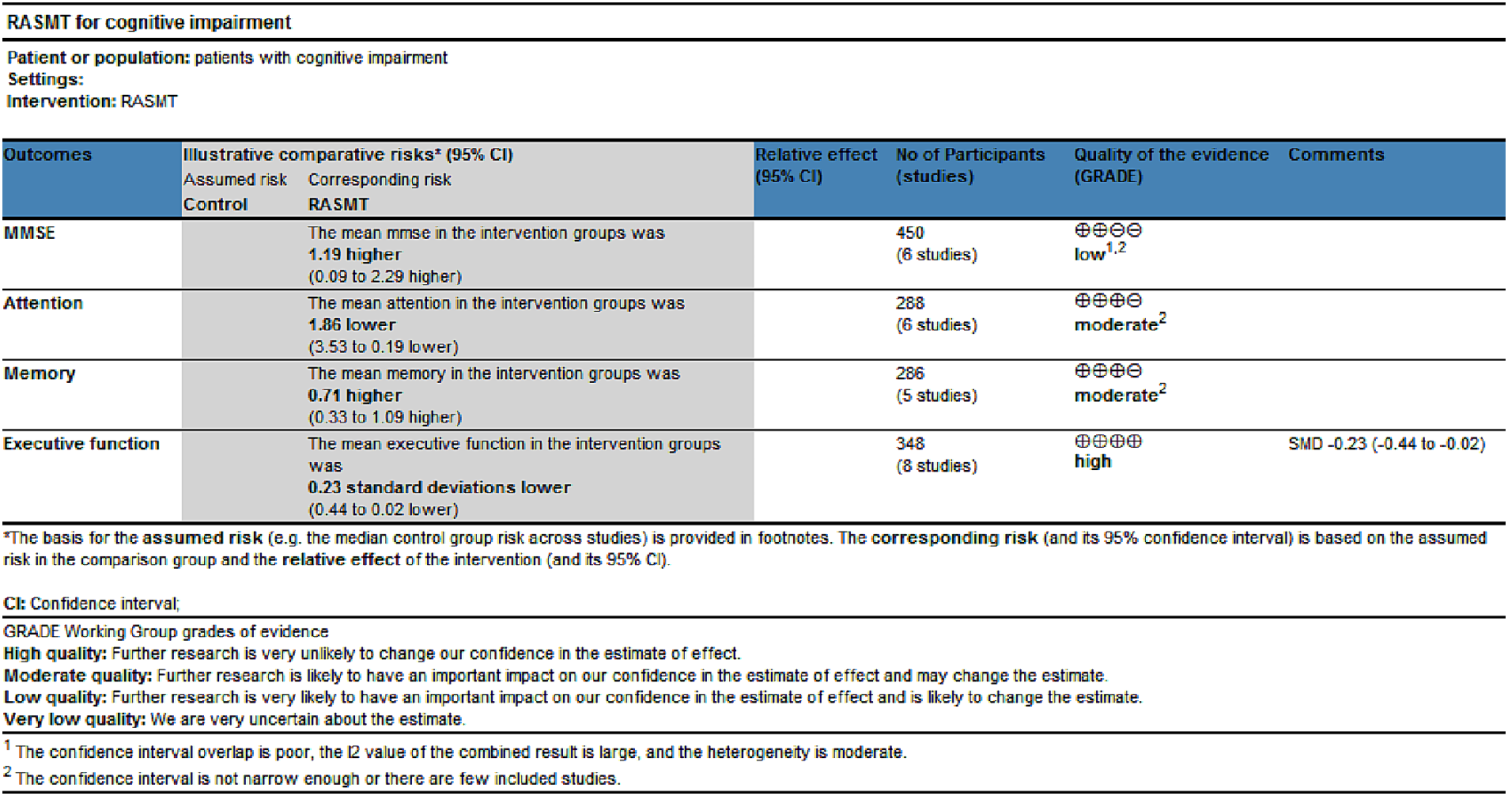
The quality of the evidence of outcome indicators.

Utilize GRADEpro GDT online tools to assess the quality of evidence for the outcome indicators integrated into the study. This research comprises four outcome indicators, with one classified as low-quality, two as medium-quality, and one as high-quality (Figure 12).

## Discussion

Epidemiological studies show that the prevalence of MCI continues to increase, and with the worsening of population aging (Chen et al., 2022), MCI will become a major risk factor threatening the health of the elderly. Short-term conversion rate from MCI to dementia is between 20 and 40%, but long-term in 5–10 years ranges from 60 to 100% (Wang et al., 2022). In addition, MCI is also a common functional disorder in PD, stroke and other diseases, the main manifestations of patients with memory, attention, executive ability, speech and logical thinking (Tatemichi et al., 1994; Li et al., 2019), seriously affect the prognosis of patients. At present, neuromusic therapy (Galińska, 2015), motor therapy (Cassilhas et al., 2007), computer-assisted cognitive training (Maggio et al., 2023) and repetitive transcranial magnetic stimulation (Kaster et al., 2018) are widely used in the treatment of cognitive impairment, and have achieved good clinical results. RASMT, in which programs follow rhythmic auditory cues (music or metronom) based on rhythmic auditory stimulation, a training method for rhythmic movements (playing percussion, playing piano, clapping, walking, etc.) of the upper, lower, and trunk, through the activation of central, peripheral to improve cognition. Therefore, this article conducted a meta-analysis of the effects of rhythmic auditory stimulation-based movement training on cognitive impairment, which suggests that RASMT can enhance the overall cognitive status, attention, memory, executive abilities, depression and anxiety in individuals with cognitive dysfunction. In addition, from the included literature, it can be seen that the RAMST program (type of exercise: play percussion instruments; time of exercise:30–60 min; frequency of exercise:2–3 times/week; duration of exercise: more than 12 weeks) was proved to be more effective in improving cognitive function.

After conducting a comprehensive analysis of six studies, our meta-analysis reveals a significant enhancement in overall cognitive status with RASMT in older people with cognitive impairment (*p* < 0.05). The selected studies primarily focused on older people with MCI (Hars et al., 2014; Doi et al., 2017; Biasutti and Mangiacotti, 2018; Biasutti and Mangiacotti, 2021; Kim and Kang, 2021), and one study involved individuals with Alzheimer’s disease experiencing mild to moderate cognitive impairment (Satoh et al., 2017). Despite variations in intervention methods across studies, all incorporated rhythmic movement training accompanied by rhythmic music. In three of the studies (Hars et al., 2014; Satoh et al., 2017; Kim and Kang, 2021), participants engaged in rhythmic movements with their upper limbs, lower limbs, and trunk, following the musical rhythm. Activities included clapping, stretching, dancing, walking, and muscle training. The intensity of the exercises gradually progressed from simple, repetitive, and slow-paced music to more complex, varied, and fast-paced music. In the other three studies (Doi et al., 2017; Biasutti and Mangiacotti, 2018; Biasutti and Mangiacotti, 2021), a training program designed by professionals in music and neuropsychology was utilized. It involved participants following rhythmic music with body or instrument percussion. Participants in these studies stimulated various cognitive skills related to movement through the imitation, creation, and execution of rhythmic patterns, encompassing aspects such as persistence, differentiation, selective and alternating attention, short-term memory, long-term memory, as well as executive functions. The conscious motor sequence encoding and planned action exercises led to an improvement in cognitive function (Street et al., 2015).

RASMT has significantly improved attention, memory, and execution functions in cognitive functions, and can be widely used in clinical. In terms of attention, the results of this meta-analysis showed that there was no heterogeneity in the results (*p* = 0.8, I^2^ = 0%) and *p* < 0.05, which was a significant difference. In the six included studies, attention was assessed in the same way, TMT-A, but in different forms of movement, including drum playing intervention with rhythmic cueing (Park and Kim, 2021), intensive cognitive music training (Biasutti and Mangiacotti, 2018), cognitive-motor dual-task training and auditory-motor synchronization training (Park and Lee, 2018), musical attention control training (Jones et al., 2021), physical exercise with music (Satoh et al., 2017), and playing percussion instruments (Doi et al., 2017). Although the forms of movement differed, they all required exact motor control and sustained attention, and subjects had to sustain their auditory attention to track the rhythmic cueing, this sustained attention caused entrainment between auditory functioning and upper extremity motor functioning, and the fast and slow music rhythms stimulated the subject’s sustained and selective attention and activated cognitive processing. For memory, the meta-analysis included five studies covering diverse populations, including older individuals with MCI (Doi et al., 2017), stroke patients with MCI (Park and Lee, 2018; Haire et al., 2021), Alzheimer’s disease patients with mild to moderate cognitive impairment (Satoh et al., 2017), and traumatic brain injury patients with cognitive impairment (Siponkoski et al., 2020). Despite variations in study populations, the meta-analysis results showed no heterogeneity (*p* = 0.63, I^2^ = 0%) and a highly significant difference (*p* < 0.01), indicating the efficacy of RASMT in improving memory across various clinical populations. In terms of executive function, some studies utilized near-infrared brain functional imaging techniques to measure changes in cerebral blood flow (CBF) levels following RASMT. The findings revealed a significant increase in CBF, particularly within the medial prefrontal cortex (mPFC) regions associated with task performance and decision-making processes. Executive function, primarily governed by the prefrontal cortex (PFC), showed improved connectivity with other brain regions, including the dorsolateral PFC (DLPFC) and sensorimotor areas (Ries et al., 2012). In individuals with MCI, the connectivity between mPFC and other brain areas has been reported to decrease; hence, RASMT may increase CBF levels, activating frontal lobe regions involved in task performance and decision-making processes. This elevated blood flow may contribute to improved functional connectivity between prefrontal cortex regions and other relevant brain networks. Moreover, studies employing fMRI techniques found that RASMT enhances executive function performance by improving connectivity between prefrontal cortex regions and other relevant brain networks (Hars et al., 2014; Shimizu et al., 2018; Siponkoski et al., 2020).

Based on our team’s conducted meta-analysis, there is no significant heterogeneity observed in memory (I^2^ = 0%), attention (I^2^ = 0%), and executive function (I^2^ = 0%) as cognitive indicators. However, overall cognitive status shows a highly significant heterogeneous effect (I^2^ = 73%). Through subgroup analysis, it was identified that the variance in intervention duration might be the source of this heterogeneity. Acknowledging this as a potential limitation in our study, we suggest that future research concentrate on investigating the impact of intervention duration on research outcomes, aiming to attain more rigorous and precise results.

### Future perspective

In the context of normal aging processes, cognitive impairments are prevalent among elderly individuals, posing a significant concern. Consequently, there is a pressing need for the exploration of effective methods to address cognitive impairments. While this study has demonstrated the effectiveness of RASMT in clinical rehabilitation of cognitive impairments based on the experiences of hospital staff, its applicability to family-based rehabilitation remains uncertain. Therefore, further research is warranted to investigate the effectiveness and safety of RASMT in family-based rehabilitation contexts. Moreover, this study lacks long-term observation and follow-up reports, leaving the sustained efficacy of RASMT unclear. Subsequent research efforts should focus on examining the long-term effectiveness and safety of RASMT in home rehabilitation settings.

Under conditions ensuring safety and efficacy, RASMT may be employed for family-based cognitive rehabilitation under the supervision of professional healthcare providers. Through regular patient evaluations and the development of personalized plans for incorporating RASMT into family-based rehabilitation, there exists the potential to enhance cognitive status and quality of life for elderly individuals, concurrently alleviating the burden on families and society at large.

### Study limitations

The literature reviewed in this study is exclusively in English, and publications in other languages have not been incorporated into our analysis. This limitation could potentially impact the comprehensiveness of our research findings. Additionally, notable variations exist in the forms of exercise (such as playing percussion, clapping, stretching, walking, etc.) and the duration of exercise involved in the research interventions. These differences in intervention methods may also introduce additional variables that could influence our research outcomes.

## Conclusion

This study suggests that RASMT can enhance the overall cognitive status, attention, memory, and executive abilities in individuals with cognitive dysfunction. Given the relative limitation in the sample size included in this study, future research should focus on expanding the sample size and employing a more rigorous study design to attain robust evidence regarding the effectiveness of RASMT in improving cognitive function.

## Author contributions

YNW: Writing – review & editing, Writing – original draft. XNW: Writing – review & editing, Writing – original draft. YC: Writing – review & editing, Writing – original draft. NX: Writing – review & editing, Writing – originaldraft. JHZ: Writing – review & editing, Writing – original draft. XH: Writing – review & editing, Writing – original draft. JPL: Writing – review & editing, Writing – original draft. PL: Writing – review & editing, Writing – original draft. JYC: Writing – review & editing, Writing – original draft. JHW: Writing – review & editing, Writing – original draft. XYS: Writing – review & editing, Writing – original draft.
